# Antibody–Drug Conjugates and Radionuclide‐Conjugated Drugs in Cancer Therapy: Mechanisms, Clinical Advances, and Future Directions

**DOI:** 10.1002/mco2.71147

**Published:** 2026-11-13

**Authors:** Chen Fu, Lifeng Yu, Li Han, Yuxi Miao, Qiuchen Chen, Zhaojin Yu, Hongxu Liu, Minjie Wei

**Affiliations:** ^1^ Pharmaceutical Sciences Laboratory Center, School of Pharmacy China Medical University Shenyang China; ^2^ Department of Pharmacology, School of Pharmacy China Medical University Shenyang China; ^3^ Department of Thoracic Surgery Cancer Hospital of China Medical University, Liaoning Cancer Hospital & Institute Shenyang China

**Keywords:** antibody–drug conjugates, combination therapy, dosimetry, molecular imaging, radionuclide‐conjugated drugs, theranostics, targeted alpha therapy

## Abstract

Targeted conjugate therapeutics link tumor‐directed vectors to cytotoxic drugs or radionuclides. Antibody–drug conjugates (ADCs) usually depend on cellular uptake and intracellular processing to deliver a pharmacological payload. Radionuclide‐conjugated drugs (RDCs) deliver radiation over an emission‐dependent range and can support imaging‐based patient selection and absorbed‐dose assessment. This review compares the two modalities through four linked areas: modular design, mechanisms of action, clinical translation, and resistance and next‐generation engineering. We examine vector pharmacokinetics, linker and chelator chemistry, payload selection, and the biological effects of beta particles, alpha particles, and Auger electrons. Randomized trials establish the clinical value of selected human epidermal growth factor receptor 2 (HER2)‐, TROP2‐, and Nectin‐4‐directed ADCs and prostate‐specific membrane antigen (PSMA)‐ and somatostatin‐receptor‐directed radioligands. Evidence for newer radionuclides, alternative targets, and combinations varies from preclinical studies to early clinical trials. The comparison separates ADC payload diffusion from physical radiation crossfire and distinguishes parent‐radionuclide chelation from recoil‐driven daughter redistribution. It also addresses imaging–therapy discordance, normal‐organ exposure, biomarkers, manufacturing, and access. The proposed framework organizes design and evidence appraisal; it is not a validated treatment‐selection model. Its practical value lies in identifying which delivery principles can be shared and which require modality‐specific validation.

## Graphical abstract

Radionuclide‐conjugated drugs (RDCs) integrate molecular targeting, quantitative imaging, and dosimetry to enable image‐guided precision cancer theranostics. Through coordinated ligand–chelator–radionuclide design, RDCs support patient selection, targeted radiation delivery, response monitoring, and adaptive treatment optimization, providing a translational framework for individualized cancer therapy.

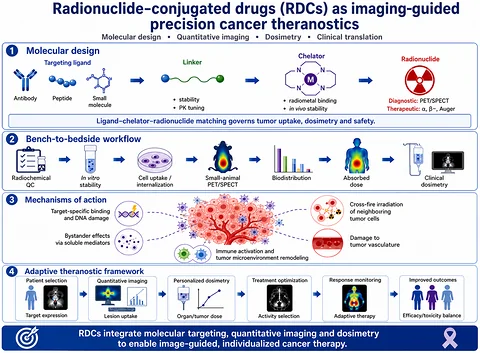

## 1 Introduction

Targeted conjugate therapeutics combine molecular recognition with delivery of a cytotoxic agent. Antibody–drug conjugates (ADCs) join an antibody to a small‐molecule payload, whereas radionuclide‐conjugated drugs (RDCs) attach a radionuclide to an antibody, peptide, or small‐molecule vector. Their clinical development has produced substantial gains in defined patient populations. The VISION and NETTER‐1 trials established the therapeutic value of prostate‐specific membrane antigen (PSMA)‐ and somatostatin‐receptor (SSTR)‐directed radioligands in metastatic prostate cancer and midgut neuroendocrine tumors (NETs), respectively [1, 2]. In parallel, the DESTINY‐Breast03 and DESTINY‐Breast04 trials showed that ADC engineering can extend benefit across human epidermal growth factor receptor 2 (HER2)‐positive and HER2‐low breast cancer [3, 4]. These successes have increased interest in comparing the delivery problems shared by the two modalities, including heterogeneous target expression, incomplete tumor penetration, and exposure of normal tissues.

The shared architecture does not imply identical requi rements. ADC efficacy depends on antigen accessibility, trafficking, linker cleavage or antibody catabolism, payload sensitivity and, for some constructs, diffusion into neighboring cells. RDC efficacy depends on the time‐dependent distribution of activity and the spatial deposition of radiation; internalization can improve retention but is not universally required. Chelator stability, emission range, and daughter‐nuclide redistribution introduce constraints that have no direct counterpart in conventional ADCs [5, 6, 7, 8]. We use RDC as an operational term for a targeting‐vector–radionuclide construct, including directly radiohalogenated vectors and radiometal conjugates containing a chelator. The broader category of radiopharmaceutical therapy also includes unconjugated agents, such as Radium‐223 dichloride, which is discussed here as a clinical comparator. Diagnostic radiopharmaceuticals inform theranostic development but should not be interpreted as therapeutic RDCs.

This narrative review compares ADCs and RDCs through four areas: modular design, mechanisms of action, clinical translation, and resistance and next‐generation engineering. We first relate vector, linker, payload, and radionuclide choices to measurable delivery properties. We then examine how these properties influence cell killing, imaging, dosimetry, clinical efficacy, and normal‐organ toxicity. Established clinical evidence is considered separately from exploratory targets, radionuclides, and combinations. The comparison is intended to identify transferable engineering principles and the limits of that transfer, including the assumptions behind diagnostic–therapeutic pairing and the evidence needed for adaptive dosing.

## 2 Modular Design of Targeted Conjugate Drugs

The components of ADCs and RDCs jointly determine distribution, retention, and therapeutic index. Changing one component can alter the performance of the entire construct. Figure 1 summarizes the structural and functional components of a radiometal‐based RDC; a chelator is not required for every radiohalogen‐labeled agent. Recent reviews have summarized the clinical development of radiopharmaceutical therapy and the bioconjugation chemistry used to build antibody‐ and radiometal‐based conjugates [9, 10, 11].

**FIGURE 1:**
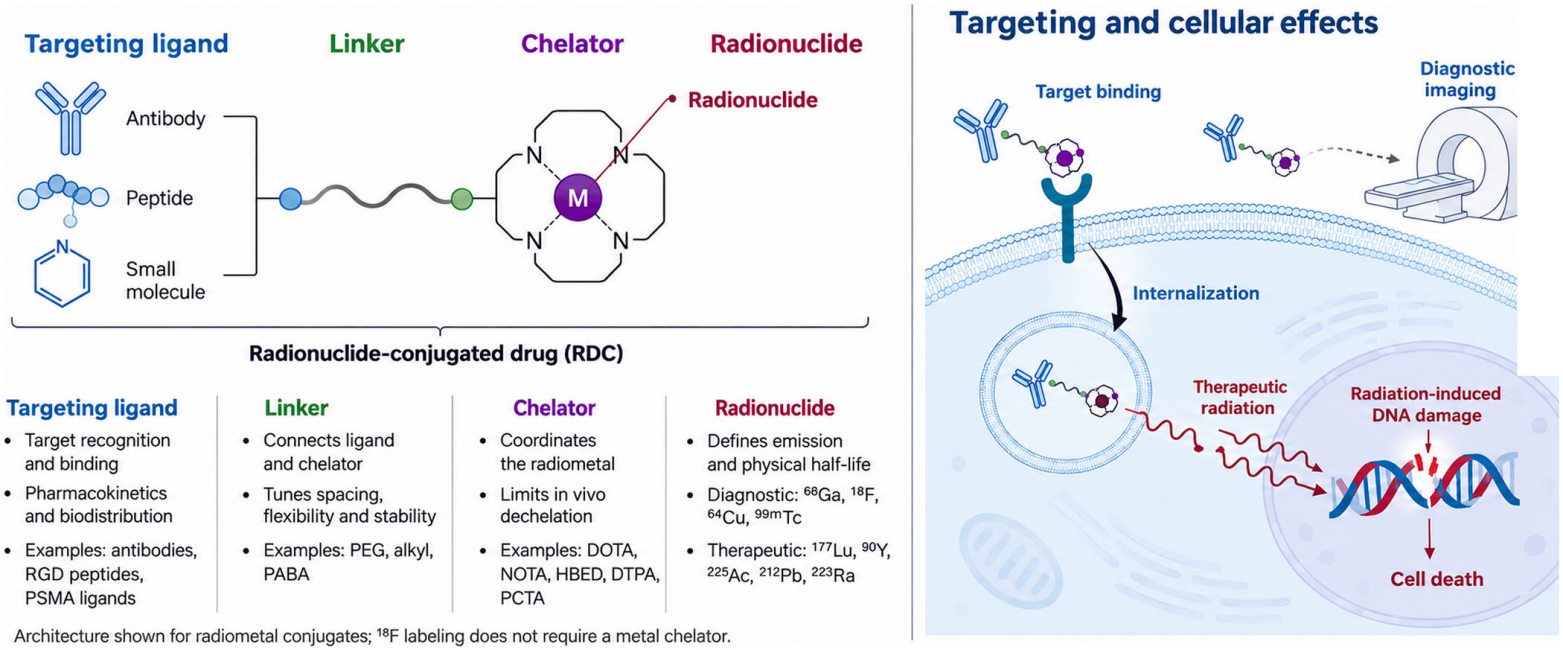
Structural composition and functional components of a radionuclide‐conjugated drug (RDC). The targeting ligand governs molecular recognition, tumor selectivity, and uptake; the linker modulates spacing, pharmacokinetics, and conjugate stability; the chelator stabilizes the radionuclide and limits dechelation or transchelation; and the radionuclide provides diagnostic emissions for PET or SPECT or therapeutic emissions for radiation‐induced cell killing. Coordinated optimization of these four modules determines biodistribution, tumor absorbed dose, normal‐organ exposure, and therapeutic index. Abbreviations: ADC, antibody–drug conjugate; PET, positron‐emission tomography; RDC, radionuclide‐conjugated drug; SPECT, single‐photon‐emission computed tomography.

### 2.1 Component Functions and Measurable Design Parameters

In a radiometal‐based RDC, the targeting vector provides molecular recognition, the linker modifies spacing and pharmacokinetics, the chelator retains the parent radiometal, and the radionuclide determines emission type, physical half‐life, and spatial energy deposition [12]. Affinity, internalization, tumor retention, serum stability, and time‐integrated activity are complementary measurements. An in vitro dissociation constant does not directly predict a positron‐emission tomography (PET) standardized uptake value (SUV) or tumor absorbed dose, because perfusion, target accessibility, administered mass, and clearance also influence uptake. In an ADC, drug‐to‐antibody ratio (DAR), conjugation site, linker stability, and payload properties jointly affect exposure and tolerability [13, 14, 15, 16]. Peptide–drug conjugates offer a related chemical‐delivery platform, although their clinical maturity and pharmacokinetics should be assessed separately from ADCs [17, 18].

### 2.2 Targeting Vectors: From Full Antibodies to Nanobodies

Targeting‐vector size affects circulation, extravasation, tissue penetration, and clearance, but these relationships are not fixed rankings of clinical efficacy. Full antibodies can provide prolonged retention while maintaining blood‐pool activity that increases marrow exposure during radionuclide therapy. Peptides and small molecules often clear more rapidly, although renal handling and receptor‐mediated retention vary substantially between constructs. The binding‐site barrier illustrates why greater affinity does not always produce more uniform penetration [19, 20]. Evaluation should therefore include internalization, residence time, lesion heterogeneity, and tumor‐to‐normal‐organ distribution alongside binding affinity. Angiogenesis‐directed and RGD‐based vectors also illustrate that the target compartment can be vascular or stromal rather than confined to tumor cells [21, 22].

Single‐domain antibodies (sdAbs or nanobodies) provide an additional vector format, typically around 12–15 kDa. Their small size can support rapid tissue access and early imaging, but renal filtration and proximal‐tubular retention remain important liabilities. Preclinical work with a ^177^Lu‐labeled anti‐HER2 nanobody showed that removal of a terminal tag and gelofusine coadministration could markedly reduce kidney uptake [23]. Development of ^68^Ga‐labeled anti‐HER2 nanobodies progressed from preclinical characterization to a phase 1 PET study; a ^131^I‐labeled anti‐HER2 VHH subsequently entered a phase 1 therapeutic study [24, 25, 26]. These studies establish feasibility for particular constructs, rather than a class‐wide safety or efficacy advantage. Radiolabeling approaches and scaffold optimization must be selected with the intended imaging or therapeutic application in mind [27, 28].

### 2.3 Linker and Conjugation Chemistry

ADC linkers must remain sufficiently stable during circulation while allowing formation of an active payload or catabolite at the intended site. Cleavable and noncleavable linkers can both support effective therapy, and intracellular antibody catabolism is important for some noncleavable constructs [6, 29]. DAR and conjugation chemistry affect aggregation, clearance, and payload exposure; site‐specific conjugation can improve these properties in selected systems [14, 15, 16]. Radiometal RDCs usually require retention of the parent radionuclide throughout delivery and decay. Their linkers can still be engineered to modify clearance or reduce renal retention, so release‐versus‐retention should be treated as a useful design distinction rather than an absolute rule for every conjugate [12, 30, 31].

### 2.4 Chelator‐Radionuclide Matching and Kinetic Inertness

Chelator selection depends on radiometal coordination chemistry, labeling conditions, kinetic inertness, and compatibility with the vector. DOTA is widely used with ^177^Lu and ^90^Y, while NOTA and HBED‐CC are established options for ^68^Ga. DFO is commonly used for ^89^Zr‐labeled antibodies, although more stable derivatives are being investigated [32]. DOTA remains prominent in clinical ^225^Ac constructs; macropa enables mild ^225^Ac labeling but its clinical maturity is lower [33, 34]. TCMC is an important chelator for ^212^ Pb platforms [35]. Figure 2 presents selected pairings and their development context rather than a ranking of every possible chelator–metal combination. Tables S1 and S2 (Supporting Information) should be read with the construct‐specific labeling and stability conditions in mind.

**FIGURE 2:**
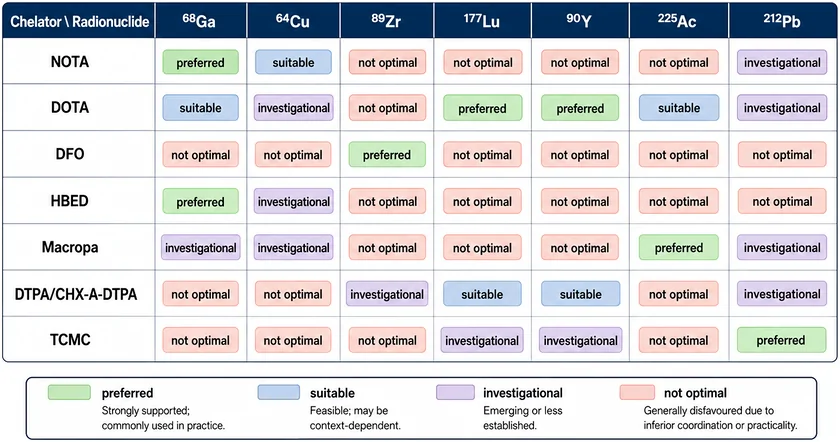
Chelator–radionuclide matching matrix for diagnostic and therapeutic RDC development. Rows list representative bifunctional chelators and columns list radionuclide classes; each cell is color‐coded as preferred (dark), suitable (medium), investigational (light), or not optimal (open) according to coordination chemistry, radiolabeling practicality, in vivo stability, and translational experience. The matrix is intended as a decision aid rather than an exhaustive catalog. Abbreviations: DFO, deferoxamine; DOTA, 1,4,7,10‐tetraazacyclododecane‐tetraacetic acid; HBED, N,N′‐di(2‐hydroxybenzyl)ethylenediamine; NOTA, 1,4,7‐triazacyclononane‐triacetic acid; RDC, radionuclide‐conjugated drug; TCMC, 1,4,7,10‐tetraaza‐1,4,7,10‐tetra(2‐carbamoylmethyl)cyclododecane.

Parent‐metal stability and daughter retention are separate problems. Alpha‐decay recoil can eject a daughter atom even when the parent radiometal complex is kinetically inert. Better chelation can reduce dechelation of the parent; it cannot prevent the nuclear recoil itself [36, 37, 38]. Intracellular sequestration and physical containment in carriers may reduce daughter escape, but their effectiveness depends on the construct, tissue, and decay chain [36, 38]. Actinium coordination chemistry, production, and radiochemical development therefore need to be assessed alongside daughter biodistribution and normal‐organ dosimetry [34, 39, 40].

### 2.5 Radionuclide Selection: Beta‐, Alpha‐, and Auger‐emitters

Radionuclide choice determines whether an RDC is optimized for imaging, therapy or theranostic pairing, and unlike a chemical payload the radionuclide acts through physical decay, emission type, particle range, linear energy transfer (LET), half‐life, and spatial dose deposition [7]. Diagnostic radionuclides must balance image quality against vector pharmacokinetics: short‐lived PET isotopes (^68^Ga, ^18^F) suit rapidly clearing peptides and small molecules, whereas longer‐lived ^64^Cu and ^89^Zr suit antibody fragments and full antibodies [41]. Figure 3 integrates this logic, connecting vector, chelator, and radionuclide selection to quantitative imaging metrics and their clinical correlates.

**FIGURE 3:**
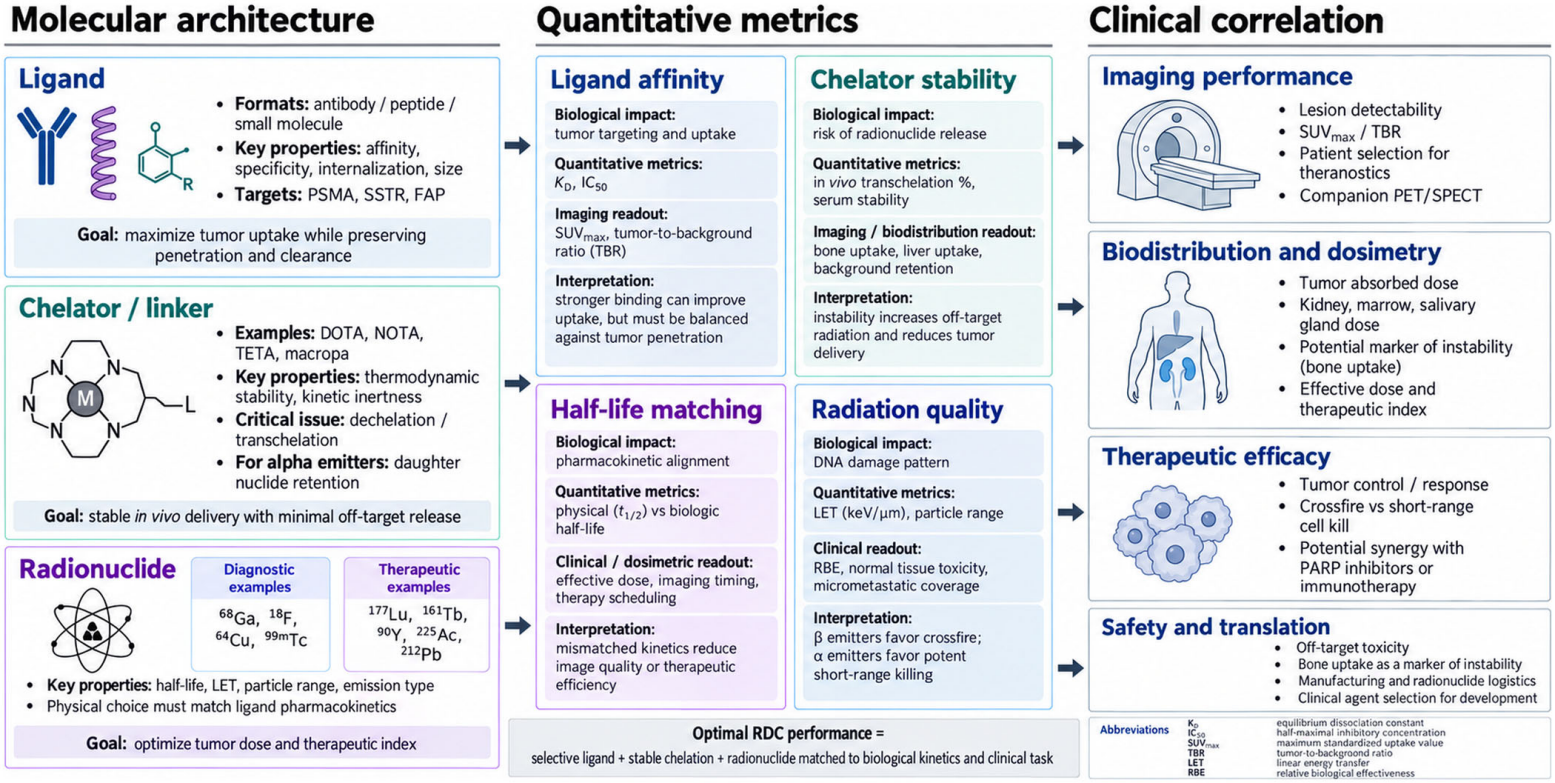
Design logic of RDCs, linking ligand, chelator, and radionuclide selection to quantitative imaging metrics and clinical correlation. Arrows indicate the sequence from molecular recognition (ligand) through radionuclide retention (chelator) to emission physics (radionuclide) and their measurable imaging read‐outs. For alpha‐emitters the logic is most stringent: high ligand affinity concentrates activity at lesions, highly inert chelators minimize recoil‐induced dechelation, and physical half‐life, linear energy transfer (LET), and particle range must match the vector's biological half‐life. Abbreviations: LET, linear energy transfer; RDC, radionuclide‐conjugated drug; SUV, standardized uptake value.

Therapeutic emission classes differ in their spatial energy deposition. Beta‐emitters such as ^177^Lu and ^90^Y provide submillimeter‐to‐millimeter crossfire and produce both single‐ and double‐strand DNA damage. Alpha radiation has a shorter range and higher linear energy transfer, producing clustered damage whose biological effects still depend on cellular and tissue context. Potential applications in small lesions or resistant disease require careful assessment of normal‐organ dose [42]. Auger‐electron effects depend strongly on subcellular localization, with DNA proximity often increasing cytotoxicity [43]. ^212^ Pb is a beta‐emitting parent used as an in vivo generator of alpha‐emitting daughters; unconjugated ^223^Ra is a comparator rather than an RDC under the structural definition used here.

The radionuclide pipeline is broadening well beyond ^177^Lu and ^225^Ac, an area of increasing interest. Terbium‐161 has emerged as a theranostic near‐analogue of ^177^Lu with a similar half‐life and beta energy but additional conversion and Auger electrons that deliver substantially higher dose to single cells and micrometastases; ^161^Tb‐PSMA‐617 outperformed ^177^Lu‐PSMA‐617 preclinically [44, 45], and the first‐in‐human VIOLET phase 1/2 trial of ^161^Tb‐PSMA‐I&T reported a favorable safety profile with a prostate‐specific antigen (PSA) decline of at least 90% in 40% of heavily pretreated patients and a median radiographic progression‐free survival of 11.1 months [46]. Thorium‐227 acts as an in vivo generator of ^223^Ra and further alpha‐emitting daughters, with the bone‐seeking property of its daughters potentially aiding retention in skeletal disease. The bone‐seeking alpha‐emitter ^223^Ra was the first alpha agent to improve survival, in the ALSYMPCA trial, establishing clinical proof of concept for targeted alpha therapy [47]. The ^212^ Pb/^212^Bi generator system permits administration of the more tractable, longer‐lived ^212^ Pb, which decays in situ to alpha‐emitting ^212^Bi at the tumor [39, 48]. Terbium‐161 has also entered SSTR‐directed use, with the first‐in‐human ^161^Tb‐DOTATOC study demonstrating feasibility [49], and ^225^Ac‐DOTATATE has shown activity in neuroendocrine tumors stable or refractory to ^177^Lu‐DOTATATE [50]. Controlled in vitro comparisons confirm the greater per‐decay lethality of alpha‐ over beta‐radiation for receptor‐targeted therapy [51]. These developments, reviewed comprehensively for the leading therapeutic radionuclides [52, 53, 54, 55, 56], motivate a radionuclide‐agnostic design logic in which emission physics, not isotope familiarity, drives selection.

### 2.6 Daughter‐Nuclide Redistribution in Alpha‐Emitter RDCs

The cytotoxic potency of alpha‐emitters is accompanied by a distinct dosimetric liability. Radionuclides with multi‐step decay chains, notably ^225^Ac, which decays through several short‐lived daughters (^221^Fr, ^217^At, ^213^Bi) to stable ^209^Bi, release recoil energy at each alpha step that can rupture the metal‐chelator bond. Once daughters such as ^213^Bi dissociate from the vector, they redistribute through the circulation and accumulate in metal‐handling organs, the kidneys, liver, and marrow, causing unintended irradiation, nephrotoxicity, hepatotoxicity, and myelosuppression [37, 38, 39].

Daughter redistribution depends on the fraction escaping the original target and the subsequent clearance and tissue affinity of each daughter. Internalization and intracellular retention may reduce escape, although the degree of retention varies with the construct and tissue. Salivary‐gland and renal exposure are important concerns in ^225^Ac‐PSMA development [42, 57, 58]. Parent‐metal stability, physical trapping in carriers, pretargeting, and decay‐chain selection address different aspects of delivery and exposure. None should be assumed to eliminate recoil‐driven daughter escape, and each requires construct‐specific validation [39].

Early clinical experience nonetheless supports feasibility. ^225^Ac‐PSMA‐617 produces antitumor activity in metastatic castration‐resistant prostate cancer (mCRPC), including in patients refractory to ^177^Lu‐PSMA therapy, with xerostomia and potential renal impairment as key dose‐limiting toxicities managed by activity titration and patient selection [42, 57, 59, 60]. ^225^Ac‐DOTATATE shows promise in neuroendocrine tumors refractory to beta‐emitting analogues. These findings indicate that, despite the theoretical risk, a clinically meaningful therapeutic window can be defined. The four design parameters introduced in Section 2—ligand affinity, chelator inertness, half‐life matching, and emission quality—thus become the concrete levers through which that window is widened, and they reappear as the interpretive axis of the mechanism, dosimetry and resistance sections that follow.

## 3 ADCs: Mechanisms and Biological Activity

ADCs commonly act through antigen binding, endocytosis, intracellular trafficking, and generation of an active payload after linker cleavage or antibody catabolism. The relative contribution of each step varies by construct [5, 61, 62, 63]. Payloads include microtubule inhibitors, Topoisomerase I inhibitors and DNA cross‐linkers. Payload potency alone does not determine clinical efficacy, because trafficking, payload efflux, cell state, and normal‐tissue exposure also influence response. Experimental comparisons confirm that antibody, payload, and cell‐line selection can each change activity [64].

Membrane‐permeable ADC payloads can reach neighboring cells after release, providing bystander killing in antigen‐heterogeneous tumors. Experiments with DS‐8201a, the precursor of trastuzumab deruxtecan, support this mechanism [65]. Bystander activity depends on the released species, its permeability and local concentration; antigen‐independent uptake or extracellular processing can contribute in some systems. A cleavable linker is therefore not an absolute prerequisite for every form of bystander activity [6]. Trastuzumab emtansine generates a relatively poorly permeable lysine‐linker‐DM1 catabolite, whereas trastuzumab deruxtecan delivers a more permeable Topoisomerase I inhibitor. Their clinical comparison reflects several pharmacological differences and does not isolate bystander killing as the sole explanation for efficacy [3, 29, 66, 67].

ADC development has improved antibody properties, linker stability, conjugation chemistry, and payload selection. Gemtuzumab ozogamicin contains a humanized antibody, and approved products differ substantially in DAR and conjugation‐site distribution. Clinical generations should therefore not be defined by a universal progression to site‐specific, homogeneous constructs. Randomized studies of gemtuzumab ozogamicin, brentuximab vedotin, polatuzumab vedotin, and inotuzumab ozogamicin, together with the single‐arm LOTIS‐2 study of loncastuximab tesirine, illustrate distinct targets and development paths [68, 69, 70, 71, 72]. Lymphoma trial landscapes and spatial profiling studies can help identify additional opportunities, but clinically investigated HER3 targeting should be distinguished from targets supported by established approvals [73, 74].

Analytical characterization is needed to distinguish total antibody, conjugated antibody, free payload, and active catabolites. Linker–payload changes can affect higher‐order structure, systemic toxicity, and nonantigen‐mediated uptake [75, 76, 77, 78]. Recent work on alternative splicing, payload choice, and dual‐payload conjugates shows why antigen expression and nominal DAR alone may not describe sensitivity [79, 80, 81]. These observations support construct‐level pharmacokinetic and pharmacodynamic assessment before a proposed design is assigned a clinical advantage.

## 4 Radionuclide‐Conjugated Drugs: Mechanisms and Biological Activity

The biological activity of RDCs derives from local energy deposition after molecularly targeted accumulation [7, 82]. Unlike ADCs, therapeutic RDCs do not require enzymatic activation or payload release; emitted radiation produces direct and indirect effects whose magnitude depends on radionuclide type, particle range, LET, subcellular localization, target density, tumor architecture, and dose rate [82, 83]. These effects—direct DNA damage, crossfire, bystander signaling, vascular injury, immunogenic cell death, and microenvironmental remodeling—are not a loose list but a graded hierarchy of certainty: direct DNA damage and crossfire are physically deterministic and clinically exploited, whereas vascular, immune, and abscopal effects are biologically plausible but not yet clinically controllable. Figure 4 depicts these mechanisms, and the subsections below are ordered from the most to the least established so that evidence level, not mechanistic appeal, guides interpretation.

**FIGURE 4:**
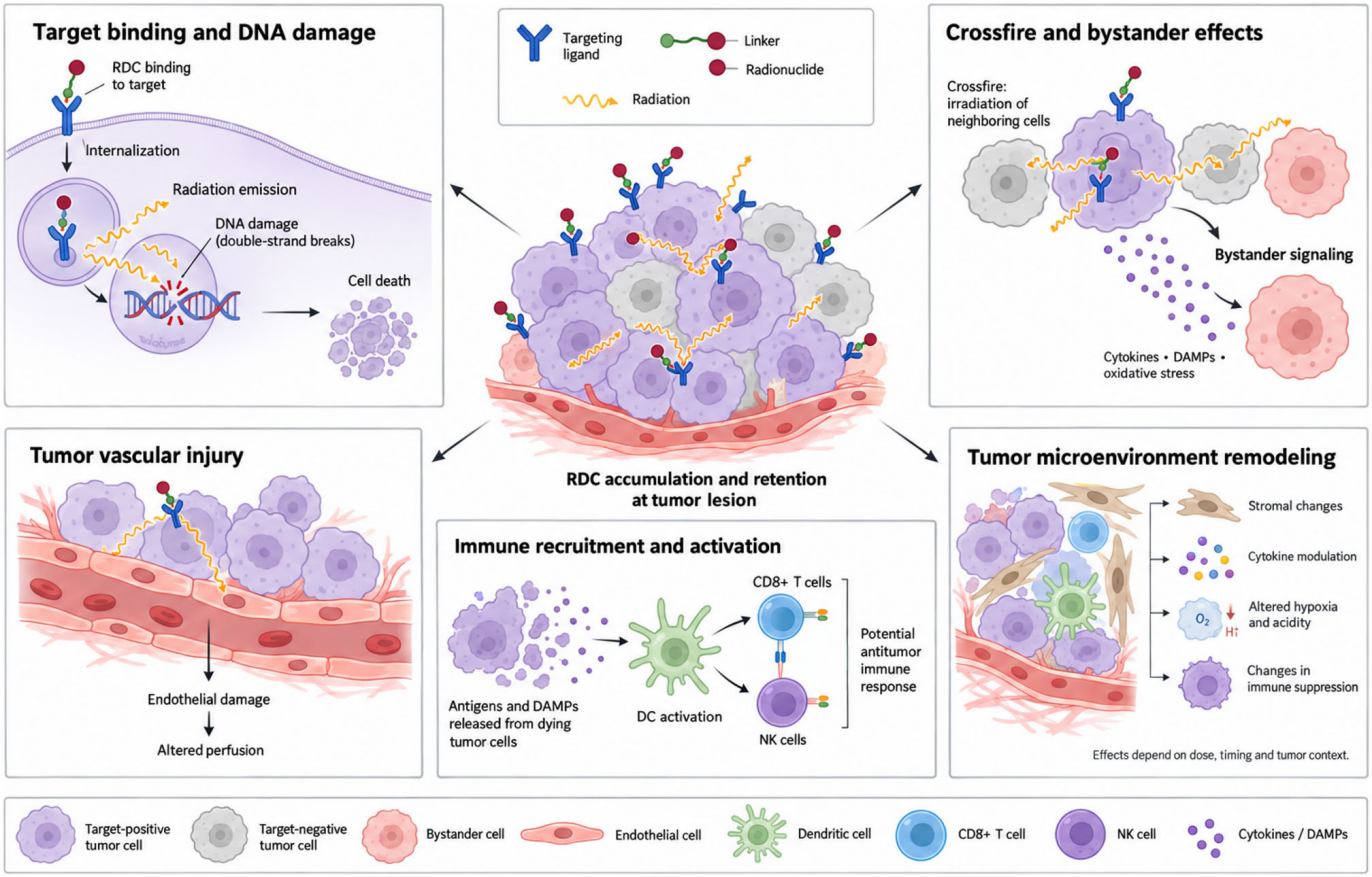
Mechanisms of action of RDCs. After target‐specific binding and retention, therapeutic RDCs deliver localized radiation that induces direct DNA damage and cell death; depending on emission type and range, crossfire irradiation reaches neighboring antigen‐low cells, and released mediators contribute to bystander effects. RDCs also damage tumor vasculature and, through immunogenic cell death, promote antigen release, dendritic‐cell activation, and cytotoxic‐immune recruitment, collectively remodeling the microenvironment and providing a rationale for combinations with radiosensitizers, DNA‐damage‐response inhibitors, and immune checkpoint blockade. Colored arrows denote radiation emissions (direct effect), radical‐mediated crossfire, and immune‐cell recruitment, respectively. Abbreviations: DNA, deoxyribonucleic acid; RDC, radionuclide‐conjugated drug.

### 4.1 Direct DNA Damage and LET Dependence

Ionizing radiation can damage DNA directly and indirectly, causing strand breaks and other lesions that impair cellular survival [82, 84, 85]. Low‐LET beta radiation produces both single‐ and double‐strand breaks. High‐LET alpha radiation generates more densely clustered lesions that may be difficult to repair, but repair dependence and treatment sensitivity remain context‐dependent. These differences motivate the study of DNA‐repair biomarkers across emission classes. For RDCs, local activity distribution and retention jointly determine energy deposition at the lesion and exposure of normal tissues.

### 4.2 Crossfire and Bystander Effects

Crossfire occurs when radiation emitted at one location deposits energy in neighboring cells. It can partly compensate for heterogeneous target expression, with the extent determined by emission range and spatial activity distribution [83, 86, 87]. Biological bystander effects instead involve signals exchanged with cells that were not directly irradiated [82]. Reactive species contribute to local indirect radiation damage; they should not be described as the physical cause of crossfire. ADC bystander killing is different again, because it is mediated by pharmacological payload diffusion.

### 4.3 Vascular and Antiangiogenic Effects

Beyond the tumor‐cell compartment, RDC‐delivered radiation also acts on the tumor vasculature, and this endothelial component is increasingly recognized as a distinct contributor to response rather than an incidental effect. High‐energy emissions injure vascular endothelial cells, triggering apoptosis and activation of the acid–sphingomyelinase–ceramide pathway, endothelial barrier disruption, and microvascular rarefaction; the resulting fall in perfusion deprives tumor cells of oxygen and nutrients and can amplify tumor‐cell killing beyond that predicted from direct DNA damage alone [88, 89]. The magnitude of this effect is dose‐rate‐ and dose‐dependent and has been characterized chiefly for external‐beam and high‐dose radiation, so its quantitative contribution during the comparatively low, protracted dose rates typical of systemic RDC therapy is uncertain and probably lesion‐specific. The vascular axis is also double‐edged: excessive vascular shutdown can create hypoxia, which lowers the oxygen‐dependent efficacy of beta‐emitters while leaving oxygen‐independent alpha‐emitters relatively unaffected—a mechanistic argument for pairing vascular‐directed strategies preferentially with high‐LET agents and a caution against uncritically combining RDCs with antiangiogenic drugs (the combination‐strategy discussion).

High‐energy emissions damage tumor vascular endothelial cells, inducing apoptosis, disrupting the vascular network, and reducing perfusion, so that oxygen and nutrient deprivation further inhibits tumor growth [88].

### 4.4 Immunogenic Cell Death and Tumor‐Microenvironment Remodeling

Radiation can increase antigen presentation and danger signaling, but it can also deplete lymphocytes or promote suppressive responses. The balance depends on dose, timing, and the tumor–immune context [90, 91, 92, 93]. Preclinical radionuclide studies, including melanin‐targeting radioimmunotherapy combined with immunotherapy, provide mechanistic support for selected combinations [94]. Conventional radiation studies of PD‐1‐targeted cytokines and irradiated brain tumors offer adjacent evidence, with exposure differences limiting extrapolation [95, 96]. Antibody‐based immunotherapy and stromal‐targeting reviews describe additional candidate pathways, but immune recruitment, acidity changes, and abscopal responses remain unvalidated treatment‐selection tools for RDCs [97, 98, 99].

### 4.5 Mechanistic Contrast Between ADCs and RDCs

The two conjugate classes therefore kill by fundamentally different logic. ADC cytotoxicity is pharmacological and cell‐autonomous‐plus‐diffusive: it requires antigen engagement, internalization and intracellular payload release, and spreads by chemical diffusion of a permeable drug. RDC cytotoxicity is physical and field‐like: it requires only proximity, does not depend on internalization for beta‐ and alpha‐emitters, and spreads by the range of the emitted particle. This distinction has direct clinical consequences. ADCs are sensitive to antigen density, internalization rate, and payload efflux; RDCs are sensitive to absorbed dose, chelator stability, and repair capacity. Consequently, resistance mechanisms, predictive biomarkers, and rational combinations differ between the classes, as developed in the discussions of clinical agents and resistance.

## 5 Clinical Translation I: Targeting‐Vector Formats and Pharmacokinetics of RDCs

Once mechanism is fixed, the first translational decision is which vector format to build on, because format—more than any other single choice—sets the pharmacokinetic envelope within which a radionuclide must act. Vector size determines circulation time, tumor penetration, the dominant route of clearance, and therefore the dominant off‐target organ, and each of these has to be reconciled with the physical half‐life of the radionuclide. RDCs are conventionally grouped by vector format into antibody radionuclide conjugates (ARC), peptide radionuclide conjugates (PRC), and small‐molecule radionuclide conjugates (SMRC); to these, the nanobody/single‐domain format introduced in the targeting‐vector discussion is added as a distinct pharmacokinetic class. The four formats are not a ranked hierarchy of efficacy but a menu of trade‐offs, and Table 1 consolidates the size, clearance, penetration, immunogenicity, dominant off‐target organ, and half‐life‐matching profile that distinguishes them; the subsections below explain why each profile arises and what it implies for radionuclide pairing.

**TABLE 1:** Comparison of antibody‐, nanobody‐, peptide‐, and small‐molecule‐based RDC targeting‐vector formats.

Feature	Antibody (ARC)	Nanobody/sdAb	Peptide (PRC)	Small molecule (SMRC)
Molecular size	∼150 kDa	∼12–15 kDa	∼1–5 kDa	Usually small; conjugate mass may exceed 1 kDa
Clearance	Slow (days–weeks)	Fast (hours)	Rapid (hours)	Often rapid; retention is construct dependent
Tumor penetration	Limited, heterogeneous	Often rapid; renal retention and distribution vary	Often rapid; distribution can be heterogeneous	Often rapid; distribution can be heterogeneous
Immunogenicity	Construct dependent; reduced by humanization	Construct dependent	Usually low; construct dependent	Usually low; construct dependent
Dominant off‐target organ	Marrow; blood‐pool exposure	Kidneys; construct dependent	Kidneys for many SSTR ligands	Kidneys, salivary glands or marrow; target dependent
Half‐life matching	Often long‐lived; matched to measured kinetics	Matched to tumor retention and clearance	Matched to measured retention; e.g., ^177^Lu	Matched to measured retention; e.g., ^177^Lu
Representative agent	^90^Y‐ibritumomab	^177^Lu/^131^I‐anti‐HER2 VHH	^177^Lu‐DOTATATE	^177^Lu‐PSMA‐617

Abbreviations: ARC, antibody radionuclide conjugate; PRC, peptide radionuclide conjugate; RDC, radionuclide‐conjugated drug; sdAb, single‐domain antibody; SMRC, small‐molecule radionuclide conjugate; SSTR, somatostatin‐receptor; TAT, targeted alpha therapy; VHH, variable domain of heavy‐chain‐only antibody.

### 5.1 Antibody Radionuclide Conjugates

ARCs use monoclonal antibodies or fragments for high‐specificity delivery and prolonged tumor exposure (circulatory half‐life of days to weeks), which favors accumulation but elevates background radiation and hematological toxicity, particularly myelosuppression [100, 101]. Their large size (∼150 kDa) limits penetration of solid tumors, favoring hematological or well‐vascularized targets [19, 20]; immunogenicity is mitigated by humanization [102]. ^90^Y‐ibritumomab tiuxetan (Zevalin) and ^131^I‐tositumomab (Bexxar) validated the approach in non‐Hodgkin lymphoma [103, 104, 105, 106], and radionuclides used with antibodies include ^131^I, ^177^Lu, ^227^Th, ^211^At, and ^225^Ac; the broader history and limitations of radioimmunotherapy are reviewed elsewhere [27, 107].

### 5.2 Peptide Radionuclide Conjugates

PRCs employ short peptides (<20 amino acids) binding overexpressed receptors such as SSTR [108, 109]. Rapid blood clearance (minutes to hours) and efficient uptake permit same‐day imaging or therapy with short‐lived radionuclides; small size (∼1–5 kDa) improves penetration and low immunogenicity is typical. ^177^Lu‐DOTATATE (Lutathera) and ^68^Ga‐DOTATATE (Netspot) are established for neuroendocrine tumors [2, 110, 111, 112]. The main liability of the PRC format is renal reabsorption of the filtered peptide, which makes the kidney the dose‐limiting organ and motivates the amino‐acid infusion protocols discussed below under organ toxicity.

### 5.3 Small‐Molecule Radionuclide Conjugates

SMRCs use low‐molecular‐weight ligands for the most rapid pharmacokinetics and deepest penetration, minimizing background but demanding very high affinity for adequate retention [1, 113, 114]. ^177^Lu‐PSMA‐617 (Pluvicto) for therapy and ^68^Ga‐PSMA‐11, ^68^Ga‐PSMA‐I&T, and ^18^F‐PSMA tracers for imaging have transformed the management of mCRPC. As with PRCs, the small size that confers penetration also drives renal and, for PSMA agents specifically, salivary‐gland uptake, so that the format's translational strengths and its dose‐limiting toxicities arise from the same physical property.

### 5.4 Comparative Summary and Approved Agents

These platforms are context‐dependent design options rather than a fixed efficacy hierarchy: antibodies give prolonged retention but higher background and marrow dose; peptides, small molecules, and nanobodies give faster clearance and better penetration but require half‐life matching and, for small scaffolds, renal‐protection strategies. Table 2 consolidates representative approved diagnostic and therapeutic agents, with explicit approval years.

**TABLE 2:** Representative approved diagnostic and therapeutic radiopharmaceuticals relevant to RDC theranostics, with approval years.

Class	Product	Radionuclide	Target	Approval year	Application/indication	References
SMRC	Pluvicto	^177^Lu	PSMA	2022 (US FDA); expanded 2025 and 2026	Therapy; selected PSMA‐positive metastatic prostate cancers; regimen depends on disease setting	[1, 115, 116, 117]
PRC	Lutathera	^177^Lu	SSTR2	2018 (US FDA); 2017 (EMA); pediatric expansion 2024 (US FDA)	Therapy; SSTR‐positive GEP‐NET; US FDA indication includes patients aged ≥12 years	[2, 110, 118]
ARC	Zevalin	^90^Y	CD20	2002 (US FDA, February)	Therapy; B‐cell NHL	[103, 119, 120]
ARC	Bexxar	^131^I	CD20	2003 (US FDA, June)	Therapy; NHL (withdrawn 2014)	[104, 105]
SMRC	Pylarify	^18^F	PSMA	2021 (US FDA, May)	Diagnosis; PCa PET	[121]
SMRC	Posluma	^18^F	PSMA	2023 (US FDA; May)	Diagnosis; PCa PET	[122, 123]
SMRC	Locametz	^68^Ga	PSMA	2022 (US FDA, Mar)	Diagnosis; PCa PET	[124, 125, 126, 127]
SMRC	Gozellix	^68^Ga	PSMA	2025 (US FDA, Mar)	Diagnosis; PCa PET	[124]
SMRC	Illuccix	^68^Ga	PSMA	2021 (US FDA, December)	Diagnosis; PCa PET	[125, 128]
PRC	Netspot	^68^Ga	SSTR2	2016 (US FDA, June)	Diagnosis; NET PET	[111]
PRC	Detectnet	^64^Cu	SSTR2	2020 (US FDA, September)	Diagnosis; NET PET	[108, 129]

Abbreviations: ARC, antibody radionuclide conjugate; EMA, European Medicines Agency; US FDA, United States Food and Drug Administration; GEP‐NET, gastroenteropancreatic neuroendocrine tumor; mCRPC, metastatic castration‐resistant prostate cancer; NET, neuroendocrine tumor; NHL, non‐Hodgkin lymphoma; PCa, prostate cancer; PET, positron‐emission tomography; PRC, peptide radionuclide conjugate; PSMA, prostate‐specific membrane antigen; SMRC, small‐molecule radionuclide conjugate; SSTR2, somatostatin receptor 2. Approval dates are indicative and jurisdiction specific and were compiled from regulatory labeling and the cited references.

## 6 Clinical Translation II: Quantitative Imaging and Dosimetry as Decision Tools

The second step of clinical translation concerns the feature that most distinguishes RDCs from ADCs: the same scaffold can be imaged and treated with, so that molecular imaging becomes a decision tool rather than a diagnostic adjunct. Bioanalysis, imaging, and dosimetry provide complementary information across development. Radiochemical quality control confirms identity, purity, molar activity, and stability; in vitro assays evaluate binding, internalization, serum stability, and metabolites [12]; preclinical PET/single‐photon‐emission computed tomography (SPECT) and biodistribution define uptake, kinetics, and organ exposure; and in the clinic, quantitative PET/SPECT with serial imaging and blood or urine sampling supports absorbed‐dose estimation, response prediction, and toxicity monitoring.

LC–MS/MS and ICP–MS support characterization of nonradioactive standards, metal content, metabolites, and degradation products, particularly alongside radio‐HPLC, radio‐TLC, gamma counting, and autoradiography [130, 131]. These must be interpreted within a dosimetric framework whose clinically relevant endpoints are tumor absorbed dose, kidney and marrow exposure, salivary‐gland uptake, lesion heterogeneity, effective half‐life, and dose–response relationships [7, 132, 133, 134]. Quantitative ^177^Lu SPECT dosimetry and PSMA‐PET acquisition are now codified in joint EANM/MIRD and EANM/SNMMI guidelines, supporting standardized, precision‐oriented practice [132, 135, 136, 137].

### 6.1 Patient‐Specific Dosimetry in Clinical Practice

A generic workflow must be grounded in real clinical dosimetry. In PRRT, kidneys and marrow are dose‐limiting, and the joint IAEA/EANM/SNMMI guidance codifies serial posttherapy imaging and blood sampling to derive organ‐absorbed doses that guide activity and cycle number [133]. Kidney‐dosimetry‐based individualization showed that many patients do not reach renal tolerance thresholds at the fixed 7.4 GBq × 4 schedule used in NETTER‐1, supporting personalized, dose‐escalated regimens in selected patients [138]. For ^177^Lu‐PSMA, dosimetry from VISION‐era and single‐center cohorts links tumor absorbed dose to PSA response while identifying salivary glands, kidneys, and marrow as the constraining organs, and supports response‐adapted retreatment [139, 140, 141, 142]. Randomized evidence from TheraP further shows that baseline quantitative PSMA‐ and FDG‐PET parameters carry predictive and prognostic information [143, 144]. Patient‐specific dosimetry thus operates on three levels: response prediction (higher tumor dose predicts deeper response), toxicity stratification (cumulative kidney and marrow dose bounds retreatment), and adaptive activity adjustment (imaging‐derived dose informs the next cycle). This transforms the fixed‐activity paradigm into a feedback‐controlled one, the quantitative core of the theranostic loop, and is the specific capability that ADCs lack.

### 6.2 Theranostic Pairing: Assumptions and Their Limits

Companion imaging underpins RDC patient selection, but its central assumption, that a diagnostic agent recapitulates the therapeutic agent's biodistribution, is not universally valid and warrants critical scrutiny. First, the diagnostic and therapeutic members of a pair often differ in radionuclide chemistry, chelator, and administered mass and dose (for example, ^68^Ga‐PSMA‐11 versus ^177^Lu‐PSMA‐617), so uptake need not predict retention or absorbed dose. Second, PET positivity does not guarantee therapeutic benefit: target expression on imaging does not ensure sufficient absorbed dose for tumor control, and discordance between PET‐avid disease and response is well documented, motivating dual PSMA/FDG imaging to exclude discordant, PSMA‐low but FDG‐avid lesions. Third, quantitative selection thresholds (for example maximum standardised uptake value (SUVmax) cut‐offs) remain poorly standardized across scanners, reconstruction methods, institutions, and trials, limiting cross‐study comparability. These caveats argue for harmonized acquisition, paired‐agent dosimetry validation and, where feasible, therapy‐isotope imaging (for example ^177^Lu SPECT, ^225^Ac surrogate imaging) rather than reliance on the diagnostic surrogate alone [145, 146, 147, 148, 149].

## 7 Key Clinically Approved and Advanced Agents

The clinical appeal of RDCs arises from combining selective targeting, radiation‐induced cytotoxicity, image‐guided selection, and adaptable dosimetry; approvals since 2016 have validated the paradigm, with PSMA and SSTR as the dominant targets [7, 8, 150]. This section reviews validated and advanced agents by target, then the biomarkers that refine selection.

### 7.1 SSTR‐Targeted Agents: From Lutathera to Targeted Alpha Therapy

Lutathera (^177^Lu‐DOTATATE) was the first US FDA‐approved peptide‐receptor radionuclide therapy, transforming management of SSTR‐positive GEP‐NETs; the phase 3 NETTER‐1 trial showed markedly longer progression‐free survival and improved response versus high‐dose octreotide [2, 110, 151]. Building on this, targeted alpha therapy is advancing: ^225^Ac‐DOTATATE shows activity in beta‐refractory NETs, and the ^212^Pb‐based agent ^212^Pb‐VMT‐alpha‐NET, designed for optimized uptake and rapid renal clearance, has shown, in a phase 1/2a study, durable disease control and objective responses with no dose‐limiting toxicities at early dose levels [48]. First‐line randomized evidence (NETTER‐2) has since extended ^177^Lu‐DOTATATE to newly diagnosed Grade 2–3 disease [118], and somatostatin–antagonist ligands may further increase tumor dose relative to agonists [152, 153]. These agents illustrate the escalation logic from beta to alpha within a single validated target.

### 7.2 PSMA‐Targeted Agents: Beta, Alpha, and Terbium Platforms

Pluvicto (^177^Lu‐PSMA‐617) gained US FDA approval in 2022 for mCRPC on the basis of the phase 3 VISION trial, which demonstrated significant gains in overall and radiographic progression‐free survival in heavily pretreated patients [1, 154, 155]. Randomized trials have positioned ^177^Lu‐PSMA‐617 against cabazitaxel (TheraP) and against androgen–receptor‐pathway‐inhibitor change in taxane‐naive disease (PSMAfore), progressively moving it to earlier lines [115, 156]. Because more than 80% of screened patients met the PSMA‐PET eligibility criteria in VISION, the agent addresses a large unmet need [1, 157]. For the substantial minority with primary resistance or relapse, alpha and terbium platforms provide next steps: ^225^Ac‐PSMA‐617 produces responses even after ^177^Lu failure, with xerostomia as the principal dose‐limiting toxicity [42, 57, 60, 158]; and ^161^Tb‐PSMA‐I&T, exploiting additional Auger emission against micrometastases, showed a favorable safety profile and a 90% PSA decline in 40% of patients in the first‐in‐human VIOLET trial [44, 46]. Together these define an emission‐tiered PSMA armamentarium.

### 7.3 Beyond PSMA and SSTR: Emerging Targets

The clinical concentration on two targets is a strategic vulnerability, and the most active frontier of RDC translation is the search for validated alternatives. Two are advancing fastest. Fibroblast activation protein (FAP), expressed on cancer‐associated fibroblasts across many epithelial and mesenchymal tumors, offers a near‐pan‐tumor target that is largely independent of tumor‐cell antigen expression; first‐in‐human dose‐escalation of the long‐retained ^177^Lu‐FAPI‐XT in 14 patients with treatment‐refractory sarcoma and other solid tumors was well tolerated with no dose‐limiting toxicity, achieving disease control in 28.6% of patients and stable disease in half of those with fibroblastic sarcomas, while confirming that improving tumor retention remains the central engineering challenge for FAP radioligands [159, 160]. Gastrin‐releasing‐peptide receptor (GRPR), overexpressed in prostate and breast cancer, is likewise entering theranostic use with antagonist peptides that can complement or substitute for PSMA when PSMA expression is low or heterogeneous [161]. Both targets extend the vector‐format and emission‐matching logic described above to new disease settings, and both illustrate that target expansion, not warhead novelty, is now the rate‐limiting step for broadening RDC benefit.

### 7.4 Landmark ADC Agents and Predictive Biomarkers

Placed alongside RDCs, ADCs occupy overlapping indications with distinct pharmacology. Trastuzumab deruxtecan has become a cross‐indication standard in HER2‐positive and HER2‐low breast cancer, HER2‐mutant lung cancer, and HER2‐expressing solid tumors, driven by its bystander‐capable DXd payload [66, 67, 162, 163]; sacituzumab govitecan (anti‐TROP2) and enfortumab vedotin (anti‐Nectin‐4) extend the class to triple‐negative breast and urothelial cancers, while trastuzumab emtansine established the paradigm and trastuzumab deruxtecan broadened it across HER2‐expressing tumors [164, 165, 166, 167, 168]. The contrast with PSMA/SSTR RDCs is instructive: ADC success has depended on payload and linker engineering to exploit antigen heterogeneity via bystander diffusion, whereas RDC success has depended on emission selection and dosimetry to exploit crossfire and to spare dose‐limiting organs. Both classes converge on the same clinical goal from opposite mechanistic directions.

Across both classes, target density (PSMA, SSTR, HER2) is necessary but insufficient to predict benefit [4, 143, 169], and multidimensional biomarkers are being sought. DNA‐damage‐repair deficiency, notably homologous‐recombination defects such as BRCA1/2 mutation and high loss‐of‐heterozygosity, may sensitize tumors to radiation‐induced damage and to poly(ADP‐ribose) polymerase (PARP)‐inhibitor combinations, a radiogenomic synthetic‐lethality rationale [170, 171, 172]. Critically, the predictive value of DDR biomarkers is emission dependent: high‐LET alpha radiation produces clustered double‐strand breaks that overwhelm and largely bypass homologous‐recombination repair, so HRR status may be less predictive for alpha‐emitters than for low‐LET beta‐emitters, for which single‐strand‐break repair is more relevant. Tumor mutational burden and neoantigen load may shape immunogenic responses and combinability with checkpoint inhibitors [91, 93]; the pretreatment immune contexture, CD8 density, M2 macrophage and alternative‐checkpoint (LAG‐3, TIM‐3) enrichment, may identify tumors needing upfront immunomodulation [95, 96]. Finally, theranostic imaging provides a dynamic functional biomarker: SUVmax, SUVmean, tumor‐to‐background ratio, functional tumor volume, and uptake heterogeneity estimate deliverable dose, and low or heterogeneous uptake predicts suboptimal response even in nominally target‐positive tumors [134, 173]. Integrating genomic, immune, and imaging biomarkers shifts selection from “does the target exist?” to “is this tumor vulnerable?”.

## 8 Resistance and Next‐Generation Engineering

The fourth framework axis examines why tumor responses to conjugate therapy are frequently incomplete and how their depth and durability can be extended. Resistance, dose‐limiting toxicity, and rational combination are treated together because they are mechanistically linked: toxicity caps deliverable dose, insufficient dose permits survival of resistant clones, and combinations aim to raise the effective differential cytotoxicity without exceeding organ tolerance.

### 8.1 Resistance Mechanisms Shared and Distinct Across Conjugate Classes

For RDCs, even high average tumor doses often fail to eradicate all clonogens because dose accumulates unevenly across lesions of differing size and vascularity, and tumor heterogeneity permits survival of poorly irradiated cells. Tumors mediate radioresistance by downregulating the target antigen (for example PSMA loss), upregulating homologous‐recombination repair, or activating ATM/ATR checkpoints [82, 170, 171]. For ADCs, resistance arises from antigen downregulation or loss, impaired internalization or trafficking, lysosomal dysfunction, and payload efflux via drug transporters. The shared node is antigen downregulation; the distinct nodes are DNA‐repair capacity (RDC) versus payload delivery and efflux (ADC). These mechanisms motivate the mitigation and combination strategies below.

### 8.2 Dose‐Limiting Organ Toxicity and Mitigation

Normal‐organ dose is the principal constraint on RDC efficacy, and the dose‐limiting organ differs systematically by emission class and vector (Table 3). Kidneys are dose limiting for renally cleared peptides and small scaffolds; salivary glands and kidneys for PSMA agents (especially alpha‐emitters); and marrow for long‐circulating antibodies and for redistributed alpha‐daughters. Established and emerging mitigations include: coinfusion of cationic amino acids (25 g lysine plus 25 g arginine, or arginine alone) that competitively block megalin‐mediated proximal‐tubular reabsorption of radiopeptides and are standard renal protection in PRRT [133, 174, 175]; alternatives such as para‐aminohippurate and gelofusine, and, for small scaffolds, molecular strategies (tag removal, albumin‐binding moieties, cleavable renal linkers) that lower kidney retention while preserving tumor uptake [23]; salivary‐gland protection during PSMA therapy (local cooling, though evidence is mixed) and consideration of radioprotectors such as amifostine; and, for alpha‐emitters, chelator inertness and daughter‐trapping to limit redistributed dose (Section 2.6). Because organ tolerance thresholds differ across platforms, patient‐specific dosimetry (Section 6.1) is the practical instrument for individualizing activity within these constraints.

**TABLE 3:** Primary dose‐limiting organs and mitigation strategies by radionuclide emission class.

Emitter class	Representative nuclides	Primary dose‐limiting organ(s)	Characteristic toxicity	Principal mitigation
Beta‐emitters	^177^Lu, ^90^Y, ^161^Tb	Kidneys or marrow; vector and schedule dependent	Nephrotoxicity, myelosuppression	Amino‐acid renal protection for SSTR PRRT; organ monitoring; personalized activity remains regimen dependent
Alpha‐emitters	^225^Ac; ^212^ Pb as an in vivo alpha generator; unconjugated ^223^Ra comparator	Salivary glands for PSMA ligands; kidneys or marrow in selected constructs	Xerostomia, renal or marrow toxicity; daughter exposure may contribute	Parent‐metal stability; activity titration; organ monitoring; daughter containment remains investigational
Auger emitters	^125^I, ^111^In; mixed‐emission ^161^Tb	Kidneys for some small vectors; otherwise distribution dependent	Subcellular dose heterogeneity; normal‐tissue retention	Localization‐aware vector design; organ dosimetry and monitoring

Absolute organ‐absorbed‐dose tolerance thresholds vary by agent, schedule and patient and should be derived from patient‐specific dosimetry and its clinical context.

Abbreviations: SSTR, somatostatin‐receptor; TCMC, 1,4,7,10‐tetraaza‐tetra(2‐carbamoylmethyl)cyclododecane.

### 8.3 Combination Strategies: A Comparative Appraisal

Because monotherapy has a therapeutic ceiling, rational combinations aim to (i) widen the therapeutic window by increasing differential cytotoxicity, (ii) radiosensitize resistant cells, chiefly by impairing DNA repair, and (iii) exploit secondary radiation‐induced immune and bystander effects [176, 177, 178, 179]. Three combination modes dominate and are compared in Table 4. RDC plus DNA‐damage‐response inhibitors is the most clinically mature: because beta‐emitters generate repairable single‐strand breaks, PARP inhibition converts these to lethal double‐strand breaks. The LuPARP phase 1 trial of ^177^Lu‐PSMA‐617 plus olaparib in mCRPC established a recommended phase 2 dose with a PSA90 response of ∼55% and manageable, mainly hematological toxicity [180], and an analogous LuPARP study of ^177^Lu‐DOTATATE plus olaparib in SSTR‐positive tumors confirmed feasibility [181]. Beyond DDR inhibition, the ENZA‐p trial showed that combining ^177^Lu‐PSMA‐617 with enzalutamide improved PSA progression‐free survival, illustrating combination with androgen–receptor‐pathway blockade [182]. RDC plus immune checkpoint inhibitors is rationally supported by radiation‐induced immunogenic cell death but clinically less mature, with efficacy contingent on the immune contexture and lacking validated biomarkers. RDC plus antiangiogenic agents is the least developed, aiming to normalize vasculature and improve delivery but risking hypoxia‐mediated radioresistance if perfusion is overly reduced. The evidence gradient across these modes, mature, emerging, and exploratory, should be stated explicitly rather than presented as equivalent.

**TABLE 4:** Comparative appraisal of the three principal RDC combination strategies.

Combination	Mechanistic rationale	Evidence level	Representative clinical data	Distinct toxicity
RDC + DDR inhibitor (PARP/ATM/ATR)	Impair repair of radiation‐induced DNA damage	Early clinical feasibility; comparative efficacy unproven	Separate PSMA‐617 and DOTATATE olaparib programs; dose and toxicity require regimen‐specific assessment	Additive hematological toxicity
RDC + immune checkpoint inhibitor	Potential immune priming after radiation; response depends on dose and tumor context	Early phase clinical evidence; regimen dependent	PSMA/pembrolizumab and DOTATATE/nivolumab studies; additive toxicity requires evaluation	Immune‐related adverse events
RDC + antiangiogenic agent	Schedule‐dependent vascular changes may improve or impair delivery	Exploratory (preclinical/early)	Limited clinical data	Hypertension, impaired perfusion, hypoxia risk

Abbreviations: ATM, ataxia‐telangiectasia mutated; ATR, ATM‐ and Rad3‐related; CTLA‐4, cytotoxic T‐lymphocyte‐associated protein 4; DDR, DNA‐damage response; DSB, double‐strand break; PARP, poly(ADP‐ribose) polymerase; PD‐1/PD‐L1, programmed cell death protein 1/ligand 1; RDC, radionuclide‐conjugated drug; SSB, single‐strand break. Evidence levels are graded to distinguish mature from exploratory approaches and should not be read as equivalent.

## 9 Translational, Manufacturing, and Regulatory Challenges

### 9.1 Ligand Discovery and Target Expansion

Clinically successful RDCs remain confined to a few validated targets (PSMA, SSTR); expansion requires high‐affinity, high‐selectivity ligands against new targets. This is constrained by the scarcity of tumor antigens that are both dispensable in normal tissue and efficiently internalized, by the cost and time of ligand optimization across antibodies, nanobodies, peptides, and small molecules, and by the affinity‐versus‐penetration trade‐off that must be reconciled with radionuclide half‐life [30]. The emerging FAP‐ and GRPR‐directed agents described above are the leading tests of whether this bottleneck can be broken: FAP promises tumor‐type breadth at the cost of a stromal target with challenging retention, whereas GRPR offers a tumor‐cell target that can complement PSMA in low‐expressing prostate cancer. Their trajectories will indicate how readily the validated design logic transfers to new antigens.

### 9.2 Supply Chain and Manufacturing

Radionuclide production requires reactors or accelerators with high capital and operating costs. ^99^Mo/^99^mTc supply is chronically fragile; clinical‐scale ^177^Lu is frequently outpaced by demand; and ^225^Ac is especially scarce, depending on limited ^229^Th stocks and complex accelerator routes, insufficient for large trials or broad adoption. Mitigation requires public–private investment in production capacity, alternative routes (accelerator‐driven ^225^Ac from ^226^Ra), portfolio diversification toward more scalable options such as ^161^Tb, and a shift from centralized toward distributed, just‐in‐time manufacture, exemplified by regional Pluvicto manufacturing facilities, to reduce decay losses and expand access [183, 184]. Concretely, the projected ^177^Lu and ^225^Ac supply–demand gap over the coming decade is expected to widen as indications expand, making capacity build‐out and ^161^Tb/^212 ^Pb adoption strategic priorities; specific numerical capacity forecasts remain vendor dependent.

### 9.3 Regulatory Pathways: RDCs Versus ADCs

RDCs and ADCs follow overlapping but distinct regulatory paths, a relevant consideration for clinical development. Both are regulated as drugs and progress through standard phase 1–3 development, but RDCs additionally require radiopharmaceutical‐specific GMP for short‐half‐life products, on‐site or just‐in‐time release, radiation‐safety and waste controls, dosimetry reporting, and companion‐diagnostic codevelopment when an imaging agent defines eligibility. ADC review centers on payload toxicology, linker stability, and often a companion antigen assay, but not on radiation logistics. Global harmonization is a particular RDC challenge: GMP standards, quality‐control specifications, and dosimetry‐reporting requirements differ across jurisdictions, complicating multicenter trials. Reimbursement and access are shaped by the high cost of both agents and, for RDCs, of associated imaging and infrastructure; value‐based pricing and real‐world evidence are increasingly used to supplement trial data and support approval and coverage decisions. These differences mean regulatory strategy, like warhead engineering, is not directly transferable between the classes.

### 9.4 Waste, Cost, and Equitable Access

RDCs generate solid, liquid, and patient‐derived radioactive waste requiring decay storage and controlled disposal under strict standards; source reduction via micro‐scale synthesis and dosimetry‐guided activity, recyclable or biodegradable carriers, and international harmonization are needed to embed sustainability [185, 186]. High R&D, manufacturing, infrastructure, and waste costs create a “theranostic divide” in which access concentrates in well‐resourced centers and high‐income countries [186, 187]. Mitigation should be concrete: value‐based and outcomes‐linked pricing; equipment leasing or installment models to lower capital barriers; single‐use automated synthesis to cut per‐dose cost; technology‐transfer and training programs; and public–private and philanthropic partnerships to establish regional production and radiopharmacy hubs in low‐ and middle‐income settings [188]. Equitable access should be a design criterion, not an afterthought.

## 10 Discussion: Scope of the Framework, Convergence, and Future Perspectives

The four‐axis framework organizes this narrative comparison of ADCs and RDCs. It has not been validated as a predictive instrument or clinical scoring system. Its purpose is to relate design choices to measurable endpoints and to distinguish established evidence from hypotheses requiring further investigation.

Targeting‐vector engineering offers opportunities for shared development, including antibody fragments and nanobodies. However, payload release, radionuclide retention, resistance mechanisms, and normal‐organ constraints require modality‐specific assessment.

First, the framework clarifies what genuinely transfers between the two classes and what does not. The targeting‐vector toolbox—including the emerging nanobody format—transfers readily, because both ADCs and RDCs must solve the same problem of concentrating a construct in tumor while sparing normal tissue. Warhead engineering does not transfer, because release and retention are opposite objectives; nor do the resistance biology, predictive biomarkers, dose‐limiting toxicities, and regulatory strategies that follow from the warhead choice. Recognizing this boundary prevents the common error of importing an ADC design intuition into RDC development, and vice versa.

Second, the framework maps the field's most consequential near‐term directions onto the specific evidence gaps each axis exposes, rather than onto generic calls for “more research.” In design, modular vector–chelator–radionuclide platforms, nanobody and pretargeting vectors, and biodegradable or daughter‐trapping carriers aim to improve pharmacokinetic control, enable repeat dosing, and reduce off‐target and redistributed dose [27, 189, 190, 191, 192, 193, 194]; complementary work on liposomal and inorganic nanocarriers that retain recoiling daughter nuclides, on nuclear‐localizing Auger‐emitter conjugates, and on hydrogel‐based and engineered living delivery systems may inform RDC delivery vehicles and Auger‐emitter nuclear targeting [195, 196, 197, 198, 199, 200, 201, 202, 203]. In mechanism, the priority validation nodes are quantifying daughter‐nuclide redistribution and its biological effects in predictive models, and establishing whether vascular, bystander, and abscopal effects are clinically actionable rather than merely observable. In translation, patient‐specific dosimetry and AI‐assisted image analysis should enable predictive, in silico treatment planning and adaptive activity adjustment, converting fixed‐dose agents into feedback‐controlled therapeutics; standardized, harmonized SUV, and dosimetry reporting is the concrete decision node that would make cross‐trial thresholds usable. In resistance, biomarker‐stratified combination trials matched to emission class—DDR‐inhibitor combinations for beta‐emitters, and alpha‐emitters for homologous‐recombination‐proficient or beta‐refractory disease—are the actionable next step, with prespecified decision criteria rather than open‐ended exploration.

Third, the framework identifies where the two modalities may converge. Emission‐ and biomarker‐matched design, dosimetry‐informed and biologically adaptive dosing, and shared vector platforms point toward a common development discipline in which the choice between a pharmacological and a radioactive warhead becomes one design decision within a unified process rather than a separation into two fields. Realizing this convergence depends on prerequisites that cut across every axis: scalable, distributed manufacturing, regulatory harmonization, and access‐oriented design, without which even a well‐validated framework cannot be delivered at scale.

Future convergence will depend on matching engineering choices to measured delivery, biological sensitivity, and normal‐organ exposure. Manufacturing capacity, reproducible measurement, and access will also influence whether promising constructs become clinically useful treatments.

## 11 Conclusion

ADCs and RDCs are the two clinically dominant targeted‐conjugate modalities, and viewing them through a single framework—modular design, mechanism, clinical translation, and resistance with next‐generation engineering—clarifies both what they share and where they diverge. They share a modular architecture and a common vector toolbox, so lessons in targeting‐ligand engineering, including the emerging nanobody format, transfer readily between them. They diverge in warhead logic: ADC linkers release a pharmacological payload that kills by internalization and bystander diffusion, whereas RDC chelators retain a radioactive atom that kills by physical emission, crossfire and, for alpha‐emitters, high‐LET clustered damage. This divergence propagates to their resistance biology, predictive biomarkers, dose‐limiting toxicities, rational combinations, and regulatory paths, none of which are directly interchangeable. The distinctive and enduring contribution of RDCs is the theranostic loop: because one scaffold can be imaged and treated with, companion imaging and patient‐specific dosimetry connect target expression to absorbed dose, response and adaptive retreatment, a capability ADCs do not possess. Realizing the full promise of both classes will require moving from empirical conjugation toward emission‐ and biomarker‐matched, dosimetry‐informed, biologically adaptive design, supported by scalable manufacturing and equitable access. The framework presented here is intended less as a summary than as an organizing instrument for that transition.

## Author Contributions

C.F., Z.Y., and M.W. conceived, designed, and revised the manuscript; C.F., L.Y., Y.M., Q.C., and L.H. wrote and revised the manuscript; C.F., L.Y., and H.L. revised the manuscript and discussed the interpretation. All authors approved the final version.

## Ethic Statement

The authors have nothing to report.

## Conflicts of Interest

The authors declare no conflicts of interest.

## Funding

This work was supported by the NSFC–Liaoning Joint Fund Key Program (U24A20816), the National Natural Science Foundation of China‐Liaoning Joint Fund, the National Natural Science Foundation of China (82304564, 82272797, 81903658), Liaoning Provincial Science and Technology Department Natural Science Foundation Doctoral Start‐Up Project (No. 2025‐BS‐0539, China), Doctoral Start‐up Foundation of Liaoning Province, Department of Science and Technology of Liaoning Province, Central Guidance for Local Scientific and Technological Development Project (2025JH2/102800061), Innovation Team Project of the Liaoning Provincial Department of Education (No. LJ222610159005), and Department of Education of Liaoning Province.

## Supporting information



Supporting File 01: mco271147‐sup‐0001‐SuppMat.docx

## Data Availability

Data sharing does not apply, as no datasets were generated or analyzed for this review.
