# Supplementary material for: Antibody–Drug Conjugates and Radionuclide‐Conjugated Drugs in Cancer Therapy: Mechanisms, Clinical Advances, and Future Directions
Source: MedComm (2020). 2026 Nov 13;7(11):e71147. doi: 10.1002/mco2.71147 (PMC13761296; doi:10.1002/mco2.71147)
Supplement: Supplementary file 1 [file MCO2-7-e71147-s001.docx]

**Antibody-Drug Conjugates and Radionuclide-Conjugated Drugs in Cancer Therapy: Mechanisms, Clinical Advances, and Future Directions**

Chen Fu ^1,2#^*, Lifeng Yu ^2#^, Li Han ^2^, Yuxi Miao ^2^, Qiuchen Chen ^2^, Zhaojin Yu ^2^*, Hongxu Liu ^3^*, Minjie Wei ^2^*

^1^ Pharmaceutical Sciences Laboratory Center, School of Pharmacy, China Medical University, Shenyang, China

^2^ Department of Pharmacology, School of Pharmacy, China Medical University, Shenyang, China

^3^ Department of Thoracic Surgery, Cancer Hospital of China Medical University, Liaoning Cancer Hospital & Institute, Shenyang, China

^#^ These authors contributed equally to this work and share first authorship.

* Correspondence: Chen Fu [(cfu@cmu.edu.cn)](mailto:(cfu@cmu.edu.cn)) | Zhaojin Yu [(yuzhaojin19830813@163.com)](mailto:(yuzhaojin19830813@163.com)) | Hongxu Liu [(hxliu@cmu.edu.cn)](mailto:(hxliu@cmu.edu.cn)) | Minjie Wei [(mjwei@cmu.edu.cn)](mailto:(mjwei@cmu.edu.cn))

Supplementary Material

**Supplementary Table 1 Common chelators for RDCs**

| No. | Chelators Name | Structure | Applicable types |
| --- | --- | --- | --- |
| 1 | DOTA |  | DOTA is recognized as one of the primary chelating agents for radioactive metal elements and is currently regarded as a "gold standard" for various isotopes, including ^111^Ln, ^177^Lu, ^86/90^Y, ^225^Ac, and ^44/47^Sc. Furthermore, DOTA has been extensively utilized in the context of ^67/68^Ga; however, it is generally considered to exhibit lower stability compared to its smaller macrocyclic derivative, DPTA. |
| 2 | DTPA |  | It is an aminocarboxylic acid chelating agent. Currently, the FDA has only approved DTPA for chelating three radioactive substances: plutonium (Pu), americium (Am), and curium (Cm). Both forms of DTPA, calcium (Ca-DTPA) and zinc (Zn-DTPA), can chelate Pu, Am, and Cm tightly. Typically, it is difficult to remove radioactive substances from the human body after they have entered it 24 hours ago, but even after days or weeks of internal contamination by radioactive substances, DTPA can still remove these radioactive substances from the body. |
| 3 | TETA |  | TETA is an octadentate N4O4 macrocyclic chelator that has only been studied in combination with ^64^Cu for radiopharmaceutical applications. Compared to the newer generations of chelators such as NOTA and CB-TE2A, TETA has less stability and kinetic inertness within the body. In addition to ^64^Cu, TETA has not been deeply researched in combination with any other radioactive metal elements. |
| 4 | NOTA |  | NOTA is a hexadentate N3O3 chelator that has been extensively utilized and is recognized as one of the most effective chelators for gallium isotopes ^67/68^Ga and copper isotope ^64^Cu. It is often referred to as the "golden partner" for Ga(^3+^) due to its favorable radiolabeling conditions and stability. Furthermore, NOTA demonstrates an exceptional binding affinity for ^64^Cu, maintaining its status as the most practical "golden partner" for this isotope, even before the introduction of novel chelators such as CB-TE2A. |
| 5 | TRAP |  | TRAP is a derivative of the macrocyclic NOTA, and compared to NOTA, its derivative TRAP-Pr shows a significantly higher specificity for Ga(^3+^). Its further derivative, NOPO, also exhibits excellent radiolabeling performance in ^64^Cu and ^68^Ga. |
| 6 | HBED |  | HBED and its derivative SHBED have already undergone several decades of development, and their bifunctional derivative HBED-CC has been coupled to antibodies and found to be faster for radioactive labeling than NOTA. |
| 7 | DFO |  | DFO is a natural Fe(^3+^) carrier within bacteria, widely used for the isotopes of gallium (Ga) and zirconium (Zr). DFO is currently the most suitable chelator for ^89^Zr. |

**SupplementaryTable 2 Classification of radioactive nuclides**

| Functions | Classification | Radioactive nuclides |
| --- | --- | --- |
| Diagnostic imaging | Positron (β+) emission nuclide | ^18^F, ^61^Cu, ^68^Ga, ^89^Zr, ^64^Cu |
|  | Single-photon-emission nuclide | ^99^mTc, ^111^In |
| Treatment | α radiation nuclide | ^225^Ac, ^223^Ra, ^213^Bi, ^211^At |
|  | β radiation nuclide | ^188^Re,^177^Lu, ^161^Tb, ^131^I, ^90^Y, ^89^Sr，^153^Sm |
|  | α, β radiation nuclide | ^212^Pb |
|  | Auger electron-emission nuclide | ^161^Tb, ^125^I, ^77^Br, ^195^mPt |
